# Successful Endovascular Bailout Strategy for Retained Accunet Embolic Protection Device During Vertebral Artery Stenting

**DOI:** 10.3389/fneur.2019.00189

**Published:** 2019-03-11

**Authors:** Suraj Sulhan, Kristopher Lyon, Walter S. Lesley

**Affiliations:** ^1^Baylor Scott and White Medical Center, Department of Neurosurgery, Temple, TX, United States; ^2^Department of Surgery, Texas A&M University College of Medicine, Temple, TX, United States

**Keywords:** stent, vertebral artery, embolic protection device, endovascular, stroke

## Abstract

**Introduction:** Vertebral artery stenosis can lead to posterior circulation TIAs and stroke. Stenting is often performed to treat symptomatic vertebral artery stenosis. As with carotid stenting, embolic protection devices (EPD) are increasingly used when stenting a vertebral artery stenosis. In general, EPDs may rarely become detached or retained in the circulation during stent revascularization. We discuss a 77-year-old male with a history of cerebral atherosclerosis and prior left occipital lobe and right insular infarcts who presented with increasing left sided weakness and was found to have severe stenosis of the proximal left vertebral artery. We report the only known case and successful endovascular bailout for an irretrievable EPD occurring during vertebral artery stenting.

**Methods:** Systematic reviews of the medical literature were performed using PubMed and multiple combinations of keywords to search for irretrievable EPDs in either the carotid or vertebral arteries. The bibliographies of the results were used to identify additional publications until this process was exhausted.

**Results:** No prior reports were found for retained or detached vertebral artery EPD. A total of six cases were found where an EPD was lost in the carotid circulation. In three of the cases, a carotid arteriotomy was required to retrieve the EPD. In two other cases, diagnostic catheters were used to retrieve the EPD. In our case, an EverFlex Biliary Stent was used to flatten the irretrievable EPD into the vertebral artery wall while preserving robust vertebral artery perfusion. 21-month clinical and 16-month imaging follow-up demonstrated durable vertebral artery patency and no ischemic symptoms.

**Conclusion:** Successful bailout strategy for a retained vertebral artery EPD during stenting may be achieved with a self-expanding stent. The resultant revascularization remained durable and without clinical sequelae.

## Introduction

Atherosclerotic disease of the vertebral arteries (VA) remains a common pathology that may frequently lead to transient ischemic attack (TIA) and stroke. VA stenosis is associated with higher rates of early recurrent stroke, with a risk profile similar to (or worse) than that for carotid disease (1). Evidence suggests that the 90-day risk of recurrent stroke is 16% in patients with extracranial VA stenosis (2). When endovascular surgery is offered for symptomatic extracranial VA stenosis, data supports that the intervention should probably be undertaken early after symptom onset (1). Embolic protection devices (EPDs) are well described adjuncts in endovascular management of carotid artery stenosis for their benefit of reducing perioperative stroke (3). The use of EPDs in VA stenosis is less reported, but is now increasingly utilized given the potential benefit. Here, we report a unique case of an elderly man who underwent VA stenting with an EPD that became detached within the VA. Literature was reviewed for all cases of EPDs lost in the circulation. This is the only reported case of a retained VA EPD, as well as the first report of an EverFlex self-expanding stent being used to successfully revascularize an irretrievably detached EPD in the circulation.

## Background

A 77-year-old male with a pertinent past medical history of cerebral atherosclerosis and prior left occipital lobe and right insular infarcts presented to our institution with increasing weakness on the left half of his body while on aspirin and ongoing aggressive medical management of his diabetes, dyslipidemia, and hypertension. Magnetic resonance angiography and pre-operative diagnostic angiography were done. These studies demonstrated patent, codominant vertebral arteries that converged on the basilar artery. Generalized, multivessel atherosclerosis was observed in the cerebrovasculature in which the worst lesion was a moderately severe (70%) stenosis of the proximal left VA. Other noteworthy findings included the presence of significant vascular tortuosity especially in the left ascending subclavian and proximal left vertebral arteries. However, the left VA stenosis was deemed favorable by established guidelines for EPD-assisted angioplasty and stenting, including vessel diameter >3.5 mm (patient's VA maximum diameter was 5.05 mm in the mid-cervical segment), and despite the proximal VA tortuosity, this was (in the senior author's [WSL] experience/opinion) not preclusive for EPD (4). The patient elected to have left vertebral angioplasty with stent placement using EPD.

### Description of Technique

**Figure 1:**
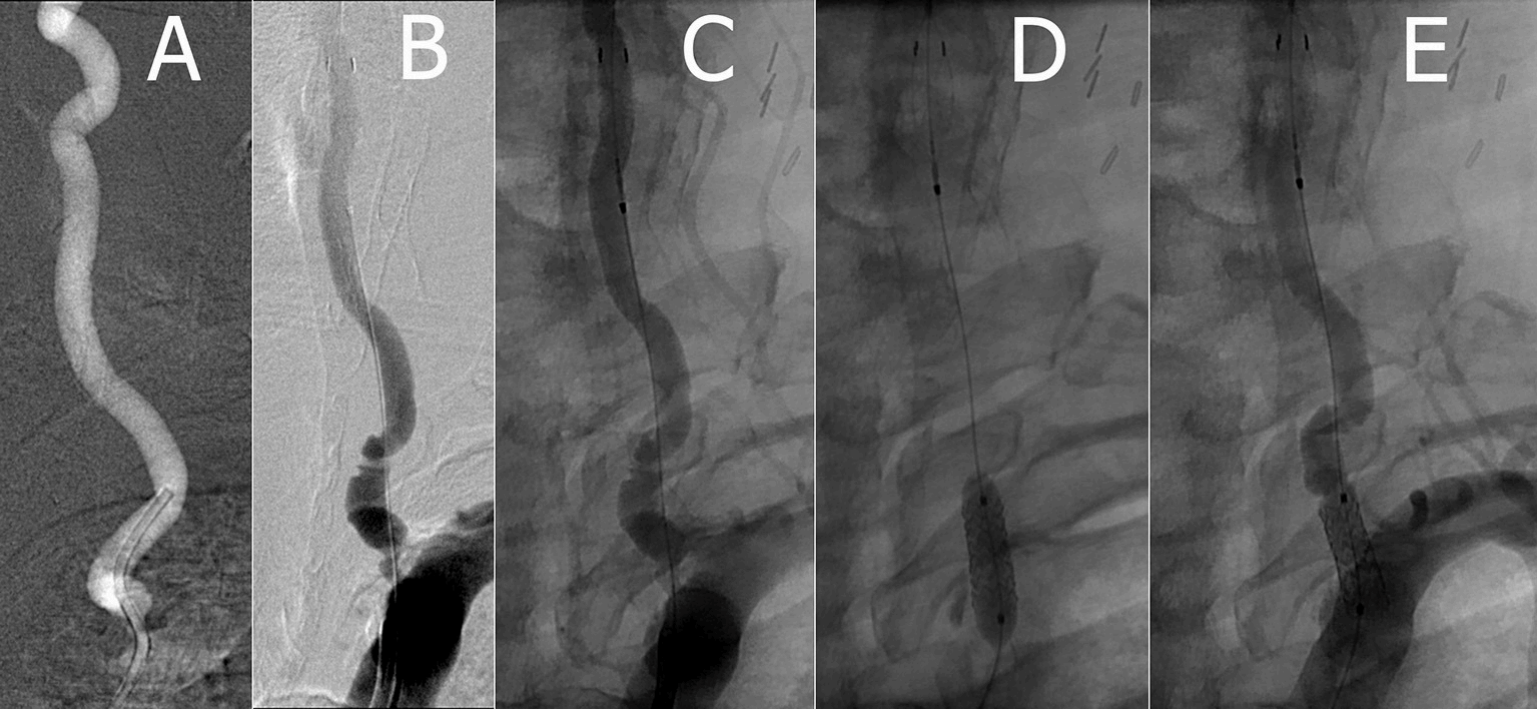
**(A)** Pre-stent left VA **(B,C)** left VA with Accunet prior to stent deployment **(D)** implanting left VA Herculink stent with balloon angioplasty **(E)** post-stent left VA.

The patient was premedicated with aspirin and Plavix. Under general anesthesia, a 6-French sheath was placed via a transfemoral access, and a 6-French Envoy guide catheter was brought up the proximal left subclavian artery. Intraoperative heparin was given to maintain an activating clotting time of 200–300 s. An appropriately sized 5.5 mm Accunet filter EPD was easily navigated through the stenotic left VA, then positioned in the mid V2 portion of the artery. A 2.5 × 12 mm NC Euphora RX balloon was positioned within the stenotic area and inflated to nominal pressure. A balloon-mounted 5 × 12 mm RX Herculink Elite Stent was then placed. Pre-implant and repeat control angiography demonstrated acceptable stent placement with subsequent prompt flow through the now resolved stenosis (Figure 1).

The Accunet filter could not be recaptured as neither the shapeable nor low-profile Accunet retrievers would pass around the angle formed by the tortuous left subclavian artery and the leading edge of the freshly implanted VA stent. Buddy wire and guide catheter repositioning were attempted to permit retriever access to the Accunet. None of these maneuvers would permit passage of the retrievers thru the stent. At this point, the filter was slowly withdrawn to see if it would simply slip thru the stent. Unfortunately, the filter indeed snagged the distal margin of the stent. The filter was readvanced free of the stent, then withdrawn but with the same result. Nevertheless, it was observed that when the partially withdrawn filter engaged the stent, the guide catheter could be advanced to the proximal margin of the stent. Using this anchor-like effect from the filter engaging the distal stent, the guide catheter was advanced over the filter wire in an attempt to pass thru the stent to recapture the filter itself. Unfortunately, during this last maneuver, the filter detached at the distal edge of the VA stent.

Antegrade flow remained remarkably robust in the left VA despite the detached EPD. This remained stable on serial angiography (15–20 min observation). During this time a quick literature search was done, but no specific guidance was found for retained VA EPD. However, a successful bailout for a carotid artery retained EPD was noted using an appropriately sized, self-expanding stent (Protégé-RX). This rescue technique seemed reasonable, and the least invasive toward offering a desirable solution. So, a 0.035” glidewire was navigated through the stent and beyond the detached filter to the mid V2 portion of the left VA. This allowed for placement of a 4-French diagnostic catheter through which an exchange length 0.035” Rosen wire was positioned. Over this wire, the catheters and sheath were removed, allowing for placement of an 8-French sheath and an 8-French NeuronMax catheter. A robust, large guide catheter was chosen to best ensure stability and full vascular access during the unpredictable bailout stent insertion and implantation.

**Figure 2:**
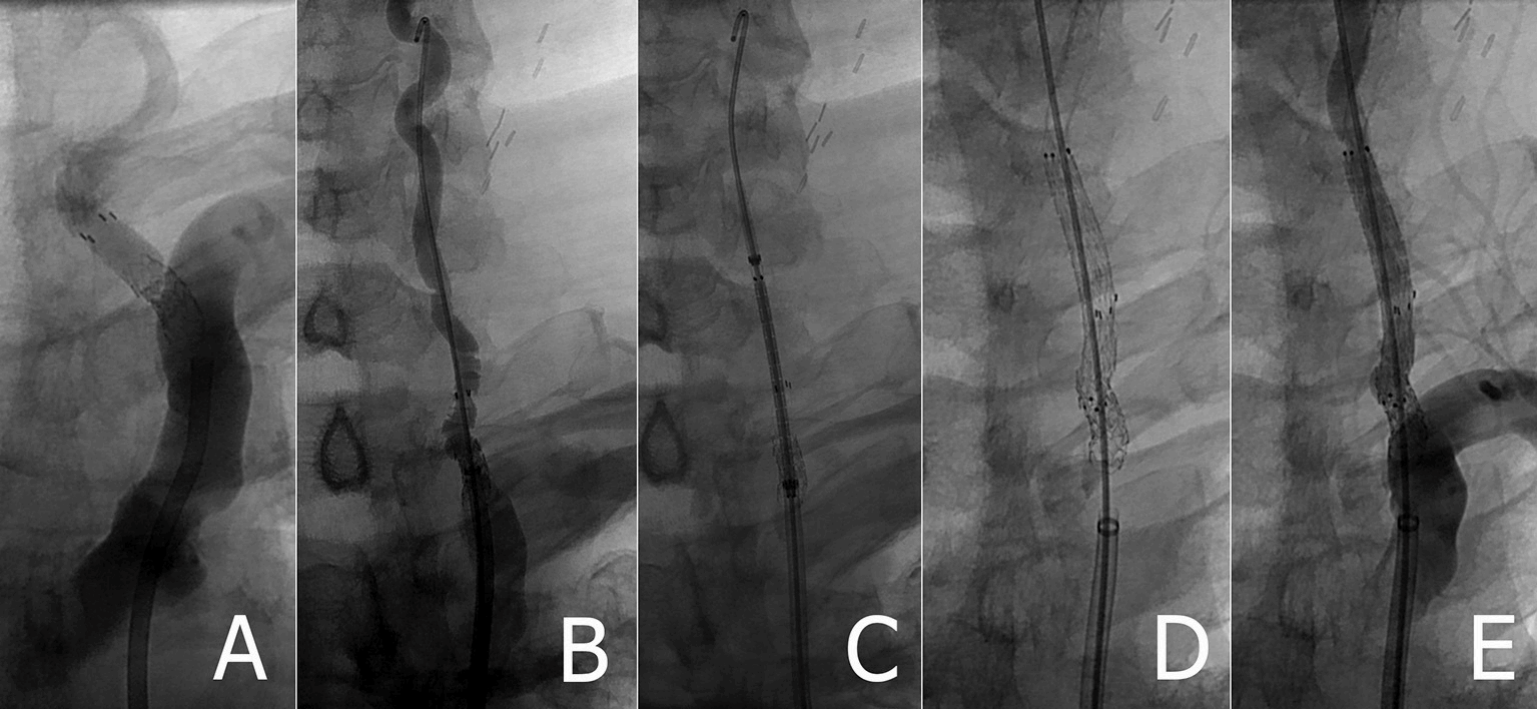
**(A)** Retained Accunet EPD following left VA stenting **(B)** Rosen wire placed following failed attempt to retrieve embolic protection device **(C)** pre-deployed position of EverFlex stent **(D,E)** left VA post EverFlex stent deployment.

A 5 × 30 mm Medtronic EverFlex self-expanding stent was chosen for four reasons: it was robust (biliary stent), appropriately sized, self-expanding, and uniformly cylindrical (unlike carotid stents that are tapered). The stent was advanced uneventfully over the wire completely covering the Accunet filter as well as overlapping the Herculink stent. The stent was implanted, which impressively flattened the Accunet filter completely against the arterial wall (Figure 2). Control angiography demonstrated robust arterial flow and no distal thromboemboli or other complication. In fact, VA blood flow appeared to be even greater that after implanting the initial Herculink stent. The wire was withdrawn and repeat control angiography showed resolution of the proximal left VA stenosis.

### Clinical Follow-Up

**Figure 3:**
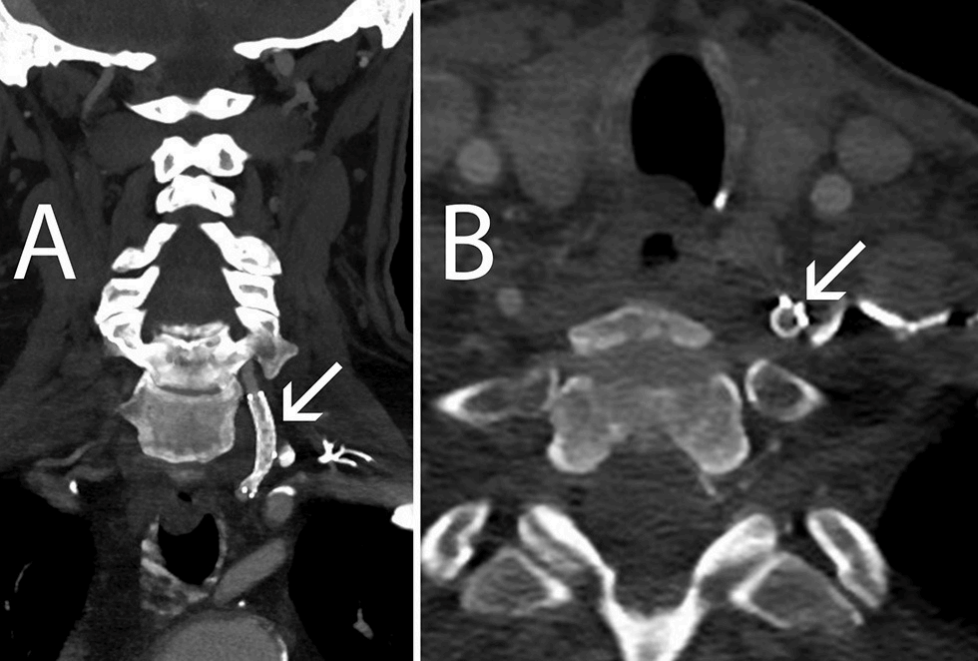
Sixteen-month follow-up CT Angiography. **(A)** Coronal view, left VA remains patent (arrow) **(B)** Axial view, the Accunet EPD (arrow) is flattened against the VA wall by the EV3 EverFlex stent.

The patient tolerated the procedure well and was discharged on 2 months of Plavix 75 mg daily and lifetime regimen of aspirin 81 mg daily. At 1-month follow-up, the patient complained of intermittent bilateral tinnitus but otherwise was neurologically stable and no bruit was present on auscultation. Sixteen month follow-up CT angiogram showed a widely patent EverFlex self-expanding stent and he has remained neurologically stable after 21 months of clinical follow-up since his VA stent surgery (Figure 3).

## Discussion

Posterior circulation strokes comprise up to 20–25% of all ischemic strokes, with an associated mortality rate as high as 30% (5, 6). It is estimated that ~20% of these cases involve atherosclerosis of the VA, frequently occurring proximally as it arises from the subclavian artery (7). This is due in part to the tortuosity, diameter, and resulting altered hemodynamic flow through the proximal VA (8). VA atherosclerotic disease is often asymptomatic due to the vast collateral blood supply, but distal lodging of atherosclerotic emboli with resulting stroke can be catastrophic (5, 9). Arterio-arterial embolism is the potential mechanism for stroke in 14 to 20% of patients who have occlusive vertebral or basilar artery disease (10). Additionally, Eberhardt et al. state that the risk of subsequent stroke over the following 5 years after a VA TIA or stroke is 22–35% (10).

Management of VA stenosis has shifted largely to endovascular surgery given the complication rate and technical difficulty of open surgery, as well as the inability of medical therapy alone to achieve long-lasting effectiveness (11). In a study with 105 patients who underwent stenting for symptomatic VA disease, 100% radiographic improvement was achieved with a 30-day 1% risk of death, and complication rate of 4.8% (12). However, in long term follow-up (median of 29.1 months), 70.5% of patients remained symptom free, and 13.1% underwent target vessel revascularization (12). Similarly, Mohammadian et al. reported a technical success rate of 97.6%, a clinical success rate of 100%, and an event-free survival rate of 92.4% in 206 patients who underwent percutaneous transluminal angioplasty with or without stent placement for symptomatic stenosis of the VA (11).

EPD use is well described in the endovascular treatment of carotid artery stenosis and is currently mandated by the Centers for Medicare and Medicaid (CMS) for reimbursement in conjunction with carotid artery stenting (3). EPDs have been shown to reduce intraoperative intracranial debris embolization and stroke (3, 5, 13). Cremonesi et al. demonstrated successful EPD use in 440 of 442 patients who underwent carotid artery stenting with no peri-procedural deaths reported and an overall complication rate of 3.4% (13). EPD use in VA stenosis is less reported given the tortuosity and smaller vessel caliber, thus making EPD use in the VA technically challenging in comparison to its use in the carotid artery (6, 14). However, the potential benefit of preventing intraoperative embolization and devastating posterior circulation stroke still exists (6). Geng et al. compared the clinical outcomes of stent placement with and without EPD use for the treatment of symptomatic atherosclerotic VA ostial stenosis in 127 patients. While their results showed a technical success rate of 95.5% (63/66 patients) in the EPD group vs. 100% (70/70 patients) without EPD use at 30 days (*p* = 0.072), a mean 18-month follow-up yielded a clinical success rate of 93.9% (62/66 patients) in the EPD group vs. 85.7% (60/70 patients) without EPD use (*p* = 0.115). Additionally, diffusion weighted imaging at 42 h post-stenting showed two hyperintense lesions in two patients (3.3%) with EPD use vs. 15 lesions in 13 patients (18.6%) without EPD use (*p* < 0.01) (5). Similar results and technical success of EPD use in VA stenting have been shown in multiple studies (6, 9, 14, 15).

To our knowledge, the present case is the only reported case of an irretrievable or retained EPD in VA stenting. However, a total of six cases of an irretrievable EPD in the carotid circulation were found and summarized in Table 1 (13, 16–18). In three of the six cases, a carotid arteriotomy was performed for stent removal. Of note, Li et al. initially compressed the EPD against the carotid artery wall prior to surgical removal (13, 17). Our case demonstrates that an endovascular approach provides an efficient and effective long-term alternative to open surgical removal of an irretrievable EPD in the VA. We used a robust EverFlex self-expanding biliary stent to completely cover and plaster the Accunet EPD to the vessel wall. By doing so, the risks associated with a vertebral arteriotomy as well as the technical difficulty in exposure were avoided. The long-term implications of leaving a device within the VA, however, is an uncertain condition, obviating a larger cohort to evaluate the safety and efficacy as well as to establish development of subsequent stent fragmentation, migration or restenosis (17, 20). With permanent intravascular device placement, dual anti-platelet therapy is recommended as well as vigilant follow-up and detailed patient education regarding the awareness of symptoms prompting medical attention and treatment.

**Table 1:** Reported cases of irretrievable embolic protection devices^*^.

**References**	**Artery**	**EPD**	**Complication**	**Treatment**
Cremonesi et al. (13)	Left CCA	7 mm Angiogard	Wire of Angiogard EPD trapped in proximal stent	CCA arteriotomy to retrieve EPD
Daugherty et al. (16)	Right CCA	6 mm Angiogard	Retained EPD; retrieval device would not advance through stent	5F 125 cm diagnostic catheter to retrieve over 180 cm Angioguard wire
Daugherty et al. (16)	Left CCA	6 mm Angiogard	Retrieved EPD; retrieval device snags distal stent	5F 125 cm diagnostic catheter to retrieve over 180 cm Angioguard wire
Li et al. (17)	Right ICA	5 mm SpiderFX distal filter	Stent migrated distally and lodged in EPD; Retained EPD and guidewire; guidewire fragments over 1 month -> emboli/aorta puncture	eV3 7 × 40 mm Protégé RX stent in the right ICA to compress the EPD, then arteriotomy to retrieve stents
Tocco-Tussardi et al. (18)	Right ICA	FilterWire EX	Retrieval sheath unable to pass through nitinol loop due to resistance; nitinol loop detached from FilterWire EX guidewire and dislocated to the proximal MCA	Attempt to mobilize the device by expanding a catheter guided balloon at the level of the FilterWire EX; interrupted due to patient manifesting left faciobrachiocrural motor hemisyndrome; subsequent right anterior lenticular nucleus and right insular-temporal cortex infarcts
Page et al. (19)	Left ICA	5-mm SpiderFX distal filter	Retained EPD; recapture device unable to retrieve EPD despite adequate stent flow	CCA arteriotomy to retrieve EPD
Present Case	Left VA	5.5 mm Accunet	Accunet detached at distal stent edge during retrieval; F/U at 16 months shows widely patent Everflex.	5 × 30 mm eV3 EverFlex Biliary Stent deployed to compress EPD into vessel wall

* *CCA, common carotid artery; EPD, embolic protection device; F/U, follow-up; ICA, internal carotid artery; MCA, middle cerebral artery; VA, vertebral artery*.

## Concluding Remarks

EPDs are increasingly being used as adjuncts in VA stenting but carry the feared complication of becoming irretrievable in circulation. An endovascular rescue can be used to flatten the EPD against the vessel wall to safely avoid migration and devastating neurological compromise.

## Ethics Statement

All procedures performed in studies involving human participants were in accordance with the ethical standards of the institutional and/or national research committee and with the 1964 Helsinki declaration and its later amendments or comparable ethical standards. All devices utilized in the embolization procedures had PMA, 510(k), or HDE approvals and were on the shelf.

Informed consent: Institutional Review Board approval and both written and informed patient consent were obtained before data was retrospectively collected. Informed and written patient consent to publish this report was also obtained.

## Author Contributions

SS, KL, and WL all made substantial contributions to the conception or design of the work; as well as the acquisition, analysis, or interpretation of data for the work. SS, KL, and WL took part in drafting the work or revising it critically for important intellectual content, and made final approval of the version to be published.

### Conflict of Interest Statement

The authors declare that the research was conducted in the absence of any commercial or financial relationships that could be construed as a potential conflict of interest.
